# The Family Mealtime Observation Study (FaMOS): Exploring the Role of Family Functioning in the Association between Mothers’ and Fathers’ Food Parenting Practices and Children’s Nutrition Risk

**DOI:** 10.3390/nu11030630

**Published:** 2019-03-15

**Authors:** Kathryn Walton, Emma Haycraft, Kira Jewell, Andrea Breen, Janis Randall Simpson, Jess Haines

**Affiliations:** 1 Department of Translational Medicine, The Hospital for Sick Children, Toronto, ON M5G 0A4, Canada; 2 School of Sport, Exercise and Health Sciences, Loughborough University, Loughborough, Leicestershire LE11 3TU, UK; e.haycraft@lboro.ac.uk; 3 Department of Family Relations & Applied Nutrition, University of Guelph, Guelph, ON N1G 2W1, Canada; kjewell@uoguelph.ca (K.J.); abreen@uoguelph.ca (A.B.); rjanis@uoguelph.ca (J.R.S.); jhaines@uoguelph.ca (J.H.)

**Keywords:** family meals, food parenting practices, preschoolers, nutrition risk, direct observation

## Abstract

This cross-sectional study explores associations between mothers’ and fathers’ food parenting practices and children’s nutrition risk, while examining whether family functioning modifies or confounds the association. Home observations assessed parents’ food parenting practices during dinnertime (*n* = 73 families with preschoolers). Children’s nutrition risk was calculated using NutriSTEP^®^. Linear regression models examined associations between food parenting practices and NutriSTEP^®^ scores. An interaction term (family functioning × food parenting practice) explored effect modification; models were adjusted for family functioning to explore confounding. Among mothers, more frequent physical food restriction was associated with higher nutrition risk in their children (β = 0.40 NutriSTEP^®^ points, 95% Confidence Interval (CI) = 2.30, 7.58) and among both mothers and fathers, positive comments about the target child’s food were associated with lower nutrition risk (mothers: β = −0.31 NutriSTEP^®^ points, 95% CI = −0.54, −0.08; fathers: β = −0.27 NutriSTEP^®^ points, 95% CI = −0.75, −0.01) in models adjusted for parent education and child Body Mass Index (BMI) *z*-score. Family functioning did not modify these associations and they remained significant after adjustment for family functioning. Helping parents to use positive encouragement rather than restriction may help to reduce their children’s nutrition risk.

## 1. Introduction

Nutrition plays a critical role in the growth, development, school readiness, subsequent academic achievement, and overall health status of young children [1]. Eating preferences and patterns are established early in life [2] and longitudinal research shows that food preferences and choices during the preschool years are strongly associated with dietary patterns and food choices later in life [3]. This stability of eating habits from early childhood to adulthood [4,5] suggests that young children’s eating habits have important implications not only for children’s current health, but also their risk of future chronic disease [6,7,8]. Despite the evidence that the preschool age is a key time for establishing healthy dietary patterns, few Canadian preschoolers are meeting dietary recommendations [9,10]. It is estimated that 11–30% have moderate risk for poor nutritional intake and 10–17% have high risk, as assessed by the Nutrition Screening Tool for Every Preschooler (NutriSTEP^®^), a validated measure used to assess eating habits and identify nutrition problems in preschool-aged children [11,12,13]. Nutrition risk can range from under- to overnutrition and, among preschoolers, is defined as the presence of characteristics that can lead to nutritional deficiencies including poor growth, under or over consumption of certain food groups, and difficulties chewing or swallowing [11].

Parents are the primary influence in young children’s lives and there is evidence to suggest that parents’ food parenting practices influence children’s dietary intake [2,14,15,16] and their resulting risk of inadequate nutrition, i.e., their nutrition risk [13]. However, existing research exploring the influence of food parenting practices has produced equivocal results. For example, although early research demonstrated that restricting access to less healthful foods was associated with higher subsequent intakes of these foods [17], results from more recent studies suggest that parental restriction may be associated with healthier dietary intakes among young children [18].

Recent scientific reviews [19,20] have identified that these inconsistent findings may be due to the methodological limitations of existing research, including a reliance on parental report to assess food parenting. The validity of such reports is limited due to potential error through inaccurate recall or bias due to social desirability. Recent research has found inverse associations between parental report and observed feeding practices [21], as well as no associations at all [22,23,24]. Therefore, direct observation is needed to more accurately explore associations between food parenting practices and children’s nutrition risk.

In addition to a reliance on parental-report measures, the current literature does not account for the feeding context, including the home environment, in which feeding interactions transpire and the presence of mothers and fathers during mealtimes. Family systems theory posits that individual or family behaviors must be understood within the global family context or system [25]. General family factors, such as family functioning, may moderate the associations of food parenting practices on children’s nutrition risk because they influence how the feeding practices are experienced by the child [26]. Family functioning is defined by how family members manage daily routines, communicate and connect emotionally with one another [26]. There is some evidence that family functioning is associated with frequency of fast food consumption among adolescent females [27]; however, the mechanisms by which family functioning influences dietary intake remain largely unknown, especially among young children.

Although mothers have traditionally held the primary role in feeding children, fathers also play an important and increasingly more prominent role during feeding interactions [28]. Unfortunately, fathers have been underrepresented in existing feeding research [28,29]. Mothers and fathers have been found to differ in the food parenting practices they use [13,30], highlighting the need to explore the potentially differential relationship between their food parenting and their children’s nutrition risk. Furthermore, the existing body of literature has been based on studies conducted outside of Canada where feeding and parenting norms may differ. To date, only two studies exploring food parenting practices among preschoolers have been conducted in Canada [13,31]; both used parental report of food parenting practices.

The overall aim of this study is to address the limitations of existing research through a cross-sectional study to examine the associations between mothers’ and fathers’ food parenting practices, assessed via direct observation in the home, and children’s nutrition risk among a sample of Canadian families with preschool-aged children. We hypothesized that: (1) the children of parents who engage in controlling and pressuring food parenting would have higher NutriSTEP^®^ nutrition risk scores and therefore poorer nutritional status than those whose parents do not use these food parenting practices; and (2) children whose parents use positive encouragement during meals would have lower NutriSTEP^®^ nutrition risk scores than those whose parents do not use these food parenting practices. The second aim of this study was to examine whether family functioning modifies or confounds the association of food parenting practices and children’s nutrition risk. We hypothesized that family functioning would moderate the association between controlling food parenting practices and children’s nutrition risk. Compared to children from families with high family functioning, the positive associations between controlling food parenting practices and nutrition risk would be stronger among children from families with low family functioning.

## 2. Methods

### 2.1. Participants and Procedure

We recruited families to participate in the Family Mealtime Observation Study (FaMOS) through a variety of methods including Facebook, posters in daycare centers, visits to library story times and word of mouth. Families were eligible to participate if: (1) they had a child between the ages of 18 months and 5 years; (2) it was typical for the family to eat meals together; and (3) parent(s) were able to speak and respond to surveys in English. In families with more than one child within our target age range, the child with the closest birthday to the date of the first home visit was chosen to be the target child; in the case of twins, a coin was flipped to randomly choose the target child.

Once families were confirmed as eligible to participate, a research assistant (RA) visited each family in their home at a time that was convenient for the family. During this visit, parent(s) provided consent on behalf of themselves and their child(ren) to participate. All children in the family provided assent to be video-recorded and the target child provided additional assent to have their height and weight measured. Where possible, in two-parent homes, both parents were asked to be home during this initial visit. The parent(s) decided on which three mealtimes over the following days would be recorded. All observations collected were of the evening meal. The evening meal was chosen as it was identified by parents as the typical “family meal” and, in two-parent homes, the time when both parents usually ate with the target child. Families were reminded that we wanted to see “typical” family mealtime experiences and that there was no reason to do anything special on the nights they recorded their meal. In cases where English was the family’s second language, families were reminded to speak English during the recorded mealtimes. Each family was provided with a video camera and tripod to record their meals and the RA assisted the parent(s) with the initial video camera set-up and placement to ensure that all family members were in-view of the recording and that their faces could be seen. The RA measured parents’ and target children’s heights using a calibrated stadiometer and weights using a calibrated electronic scale. Following the home visit, we emailed parents a link to a 15-min online questionnaire, which asked them to report on their food parenting practices and aspects of their home environment, and to complete a nutrition risk questionnaire for the target child. In two parent families, each parent was sent an individual link to the questionnaire to complete.

On the evenings that a family was scheduled to record, an RA called or texted the family 15 min prior to the scheduled start of their mealtime to remind them to turn on the camera. Families were asked to record their entire meal. Study staff was not present during mealtime recordings. One hour after the family meal, an RA called or texted the family to confirm that they were indeed able to record, and to note any atypical events that may have occurred during the meal (i.e., target child had an atypical temper tantrum during the meal, camera ran out of battery, etc.). If the family was unable to record, another date was chosen for recording.

Following the recording of the three meals, an RA visited the family home again to pick up the camera. During this second home visit, the RA confirmed that three observations were recorded on the camera and that the parent questionnaires had been completed. Families were provided a $50 grocery gift card for participating. This study was approved by the University of Guelph Research Ethics Board (REB#14OC033).

### 2.2. Measures

Observed food parenting practices: we used the Family Mealtime Coding System (FMCS) [23,32] to measure mothers’ and fathers’ feeding practices. The FMCS was created to reflect the Child Feeding Questionnaire [33] and seven of its subscales were used in the current study: pressure for target child to eat (“eat three more bites”), physical prompts for target child to eat (spoon feeding child or putting food on the utensil for child to pick up), verbal restriction of target child’s food consumption (“you can’t have any more”), physical restriction of target child’s food consumption (moving a specific food away from child), food rewards to encourage target child to eat (“if you eat your peas, you can have ice cream”), non-food rewards to encourage target child to eat (“if you finish your meal, you can watch TV”), and positive comments about food (either comments about food in general, the parent’s own food, or the target child’s food, explored separately) [23]. All instances of each food parenting behavior were logged and frequencies of occurrence for each behavior were calculated for the mealtime observation. The FMCS has been found to be a reliable measure of food parenting practices among preschool aged children (inter-rater reliability >86.5%) [23,32].

Target Child’s Nutrition Risk: we measured the target child’s nutrition risk using the Nutrition Screening Tool for Every Preschooler (NutriSTEP^®^) [11]. NutriSTEP^®^ was developed by registered dietitians and is a 17-item questionnaire used to assess eating habits and identify nutrition problems in preschool aged children (3–5 years) across five subscales: eating behaviours, dietary intake, parental concerns about food and activity, screen time duration, and the use of supplements [11]. The primary parent (parent who completed the questionnaire first) completed the NutriSTEP^®^ questionnaire for the target child. NutriSTEP^®^ has been validated against registered dietitians’ assessment of children’s nutritional status (based on medical and nutritional history, three-day food records and anthropometric measurements) among a large sample of ethnically and geographically diverse families in Canada [11]. Scores on NutriSTEP^®^ and registered dietitians’ ratings were correlated (*r* = 0.48, *p* = 0.01) and the questionnaire was found to be reliable when completed by parents on two separate occasions, 2–4 weeks apart (kappa >0.75) [11]. Individual questions are answered using a tailored Likert scale, which are then coded into a numerical score through the use of the designated NutriSTEP^®^ coding system. These scores are then summed to generate a nutrition risk score for the target child (ranging from 0 to 68), with higher scores representing greater nutrition risk. For descriptive purposes, scores were tabulated to determine three levels of risk of poor nutrition based on the standardized NutriSTEP^®^ cut-offs: low (scores ≤20), medium (scores 21–25), and high (scores >25) [11]. A medium score identifies a level of risk that can be managed by public health services and a high score indicates risk requiring further assessment by a registered dietitian. For regression analyses, NutriSTEP^®^ scores were explored as a continuous variable.

### 2.3. Covariates and Moderating Variables

Parental Educational Attainment: as an indicator of socio-economic status, mothers and fathers reported their educational attainment individually on an eight-point Likert Scale ranging from eighth grade or less to Postgraduate Training or Degree. Responses were dichotomized to graduated college/university and less than a college/university education.

Child Body Mass Index (BMI): trained research assistants measured children’s heights and weights during home visit 1, as described above. Based on the World Health Organization (WHO) growth charts, we calculated BMI *z*-scores using WHO Anthro Software (version 3.2.2, WHO, Geneva, Switzerland) to assess child weight adjusted for age and gender.

Family Functioning: we measured family functioning using the “general functioning” subscale of the McMaster Family Assessment Device (FAD) [34]. Because family functioning is a family-level variable, we used the primary parent’s report of family functioning. The scale consists of statements about families to which participants indicated the degree to which they agreed on a four-point scale (strongly disagree to strongly agree) and includes items that measure the overall health/pathology of the family relating to six dimensions of family functioning: (a) problem solving; (b) communication; (c) roles; (d) affective responsiveness; (e) affective involvement; and (f) behavioral control. The FAD has been validated against experienced family therapists’ clinical ratings and found to correspond with clinicians’ ratings of healthy and unhealthy families; ‘general functioning’ subscale (t (39) = 2.49, *p* < 0.05) [35]. Test-retest reliability has also been reported for the ‘general functioning’ subscale when completed by participants one week later (*r* = 0.71) [35]. We averaged responses to each of the 12 items comprising the ‘general functioning’ subscale to create an overall score; higher scores indicate lower levels of family functioning [34]. In our sample, the Cronbach’s alpha for this subscale was 0.86, indicating strong internal consistency. For descriptive purposes, a cut-off of 2.17 was used to distinguish high and low functioning families (<2.17 = high functioning, ≥2.17 = low functioning) [35]. For regression analyses, family functioning was explored as a continuous variable.

### 2.4. Data Analysis

Videos of the mealtime observations were coded using Noldus Observer XT 12.5 (Noldus Information Technology, Wageningen, The Netherlands). To reduce camera reactivity, only mealtimes two and three were coded; video one was used as a warm-up for families [23]. Videos were coded by two independent coders who received a standardized training (4 h in length) outlining use of the Observer XT software and the behaviors to code using the FMCS [23,32]. Prior to coding videos for the current study, coders were provided with five sample videos and were required to demonstrate reliability (kappa >0.8) in comparison to pre-determined codes. During analysis, coders met bi-weekly to discuss coding challenges and to code a video together to ensure adherence to the coding scheme. Detailed notes from these meetings were kept to document decisions. Both coders also conducted regular reviews of their own coding to ensure intra-rater reliability during the coding of the full sample. A random sample of 20% of the videos were coded by both coders to ensure reliability [36]. We calculated intra-rater and inter-rater reliability using Observer XT’s reliability function. Our inter-rater reliability (kappa = 0.8) and intra-rater reliability (kappa = 0.82) were both excellent [37].

Analyses were run separately for mothers and fathers, as previous research has suggested differences in mothers’ and fathers’ food parenting practices [28]. For both the mothers and fathers, there were no statistically significant differences in food parenting practices used in videos 2 and 3; thus, scores for parenting practices were averaged across the two observations. We calculated descriptive and frequency statistics for each food parenting practice to understand the practices used during mealtimes, as well as Mann-Whitney U-tests of difference between mothers’ and fathers’ food parenting practices. We ran linear regression models to examine the association between food parenting practices and children’s NutriSTEP^®^ risk scores. To examine whether family functioning modifies the association between food parenting practices and NutriSTEP^®^ risk scores, we ran linear regression models including an interaction term, created using centered variables (family functioning × food parenting practice). There was no evidence of modification by family functioning (*p* > 0.05 for the interaction terms; results not shown); thus, we then included family functioning in the model as a covariate to examine whether family functioning confounds the association between food parenting practices and NutriSTEP^®^ risk scores. Based on previous research, all models were adjusted for parent educational attainment and child BMI *z*-score [38,39]. Additionally, we explored whether associations between food parenting practices and child nutrition risk score differed by child gender; results from stratified models showed no difference by child gender (results not shown). Statistical analyses were conducted using SPSS version 25 for Mac (PASW, IBM, New York, NY, USA).

## 3. Results

### 3.1. Participant and Family-Level Characteristics

Of the families who expressed interest in our study by completing our eligibility screener (*n* = 112), two were ineligible because they did not have a child within our target age range, three lived too far away for RAs to conduct home visits, one indicated that they did not speak English during mealtimes and 29 of the families did not follow-up to schedule a home visit. We scheduled and completed home visits with 77 families. We were unable to obtain video data from four families; one family found that the camera was too distracting for the target child, and three families spoke languages other than English during their meal recordings.

Thus, our final sample for analysis consisted of 73 families (137 parents; 74 mothers, 63 fathers). Most of the parents (*n* = 73) identified as the “primary parent” (by completing the questionnaire first) were mothers. The majority of parents in this study were married or living with a partner (94.5%; Table 1), 85.3% of parents reported having graduated from College or University or more (i.e., post-graduate degree), and 86.1% of families reported a total household income of ≥$50,000/year. Moreover, the majority of parents identified as “white” (84.6%). Most of the families in this study participated in family dinners seven days a week (83.6%) and 90.4% were considered to have high family functioning (scores <2.17).

The average age of the target child was 3.3 ± 1.1 years and 56.2% were female (Table 2). The majority of children (65.3%) were considered to have a healthy weight status and low nutrition risk (89.0%).

### 3.2. Exploration of Mealtime Videos and Food Parenting Practices

The average meal length was 24.5 min. Our results suggest that, overall, mothers were more engaged in food parenting than fathers (Table 3). Mothers used significantly more controlling feeding practices (described as mean frequency of occurrence, times/meal); specifically, more verbal restriction (mothers: 1.31 times/meal ± 1.59; fathers: 0.63 times/meal ± 0.88; *p* < 0.05), food rewards (mothers: 0.75 times/meal ± 1.79; fathers: 0.49 ± 1.13, *p* < 0.05), and non-food rewards (mothers: 0.31 times/meal ± 0.57; fathers: 0.14 times/meal ± 0.36, *p* < 0.05) than fathers. Additionally, mothers used more positive encouragement, specifically towards the target child’s food during meals, than fathers (mothers: 7.12 times/meal ± 5.31; fathers: 4.26 times/meal ± 3.45, *p* < 0.05).

### 3.3. Exploration of Food Parenting Practices and Target Child’s NutriSTEP^®^ Risk Scores

#### 3.3.1. Mothers

Among mothers, more frequent physical restriction of food was associated with higher NutriSTEP^®^ nutrition risk scores in their children (β = 0.40 NutriSTEP^®^ points, 95% CI = 2.30, 7.58) and more frequent positive comments about the target children’s food were associated with lower nutrition risk (β = −0.31 NutriSTEP^®^ points, 95% CI = −0.54, −0.08) in models adjusted for parent educational attainment and child BMI *z*-score (Model 1; Table 4). These associations remained significant after adjustment for family functioning (Model 2; Table 4). No other significant associations between mothers’ food parenting practices and the target child’s nutrition risk scores were found.

#### 3.3.2. Fathers

Among fathers, more frequent positive comments about the target children’s food were associated with lower nutrition risk (β = −0.27 NutriSTEP^®^ points, 95% CI = −0.75, −0.01) in models adjusted for parent educational attainment and child BMI *z*-score (Mode 1; Table 4). The association remained similar, but not significant (CIs just overlapping 0) after adjustment for family functioning (Model 2; Table 4). Similar to our results with mothers, adjustment for family functioning did not otherwise change our results, and no other significant associations between fathers’ food parenting practices and the target child’s nutrition risk scores were found (Model 2; Table 4).

## 4. Discussion

In this sample of Canadian parents of preschool aged children, we observed that mothers’ use of physical restriction was associated with higher nutrition risk for their child and both mothers’ and fathers’ use of positive comments towards the target child’s food was associated with lower nutrition risk. Family functioning did not modify or confound these associations. To our knowledge, this is the first study to explore the associations of observed food parenting practices with preschool children’s nutrition risk while considering the potential influence of family functioning on these associations.

Our finding that maternal use of physical restriction of food was associated with greater risk of nutritional inadequacy supports our hypothesis that controlling food parenting practices would be associated with increased nutrition risk. This finding is consistent with previous longitudinal research that suggests restriction undermines a child’s ability to recognize their own hunger and satiety cues [40] and increases eating in the absence of hunger [14], thereby increasing the child’s risk for overeating and potential for increased nutrition risk. Similar to previous observational studies, we observed fairly low levels of parental restriction [22,23,30,41,42]. It has been suggested that overt restriction (i.e., taking food away from a child or telling them to stop eating a certain food) may occur more often during less structured eating occasions such as snack time and that covert restriction may occur more during structured mealtimes (i.e., not bringing certain foods into the home or offering them at mealtimes) [43]. Future research should explore the association of restriction and children’s dietary intake and eating behaviors throughout the day using a longitudinal study design to help tease out the directionality of the association. It is possible that the mothers in this sample were concerned about their child’s nutritional intake and thus used more physical restriction during mealtimes.

Our finding that mothers’ and fathers’ positive comments about the target child’s food were associated with lower nutrition risk among children also supports our initial hypotheses. Research by Holley and colleagues [44] found parental modeling and positive comments to be helpful in increasing children’s consumption of disliked vegetables. Positive encouragement, without pressure, has also been related to lower reported levels of fussy eating and greater reports of food enjoyment in preschoolers [45]. Taken together, these results suggest that positive parental comments may be an effective method of both increasing intake of healthful foods and decreasing nutrition risk scores among preschool aged children. Future research should test the ability of interventions to teach parents to use positive encouragement rather than controlling feeding practices to improve dietary intake and reduce nutrition risk among preschoolers.

It has been argued that family dysfunction may diminish the impact of positive role modeling and intensify adverse impacts of controlling feeding practices during mealtimes [46]. However, our results suggest family functioning does not modify or confound the association between parents’ food parenting practices and preschoolers’ nutrition risk. Contrary to our hypotheses, the associations between controlling food parenting practices and greater nutrition risk were not stronger among children from families with low family functioning. Similarly, effect estimates did not change when family functioning was added to the analytic models. When it comes to reducing children’s nutrition risk, our findings suggest that food parenting practices are an important avenue of intervention, regardless of level of family functioning. However, future research should seek to replicate our findings in populations with diverse levels of family functioning; it is possible that the limited variability of family functioning in our sample may have contributed to the null effects of family functioning on the associations explored.

Mothers’, but not fathers’, use of physical restriction was associated with children’s nutrition risk. However, this finding is contrary to previous research using mother and father reports of food parenting. Among a sample of Canadian parents, Watterworth et al. [13] found that fathers, but not mothers, reported ‘restriction for health’ was associated with higher nutrition risk among children. Comparisons between the two studies are difficult due to Watterworth and colleagues’ [13] use of parental report of food parenting practices, as previous research has found little association between observed and reported food parenting practices [21,22,23,24]. Mealtime observations only capture overt restriction and parents may use covert restriction, which would not be observed during mealtime videos, but can be captured in food parenting questionnaires. Future research should explore the use of mixed observational and parent-report methods in an attempt to more accurately capture both overt and covert restriction as well as the meaning behind the restriction (e.g., for health or weight control) [47].

We found that mothers used significantly more verbal restriction and food and non-food rewards than fathers during meals. Overall, we found that positive comments about the target child’s food (e.g., “your broccoli looks delicious”) was the most commonly used food parenting practice among both mothers and fathers, followed by verbal pressure to get the child to eat (e.g., “eat three more bites”) for mothers and positive comments about food in general (e.g., “milk helps us grow strong bones and teeth”) for fathers, which is similar to previous observational research [30]. Our findings suggest that mothers and fathers differ in the food parenting practices they use. While observational studies including fathers have been limited [28], the research exploring differences in mothers’ and fathers’ observed food parenting practices has produced conflicting results [23,30]. One U.K.- based study that used the FMCS [23] reported no significant differences in the frequency of mothers’ and fathers’ food parenting practices. However, similar to our findings, an American study by Orrell-Valente and colleagues [30] found that, overall, mothers used significantly more food parenting practices than fathers and they also tended to use praise more frequently. Interestingly, the study that showed no difference between mothers’ and fathers food parenting [23] was conducted in the U.K., suggesting that food parenting practices could be regionally and culturally defined. These inconsistent results highlight the importance of exploring both mothers’ and fathers’ food parenting practices to further understand how parents may influence each other’s food parenting practices and how this interaction between mothers and fathers may impact children’s dietary intake. Harris et al., have pioneered work in this area and found that, compared to discordant mother-father pairs, Australian mother-father pairs who concordantly report low levels of pressure to eat had children with lower levels of pickiness [48]. Additional research is needed to understand the impact of concordance/discordance between mothers’ and fathers’ food parenting practices in Canada.

Our study had a number of key strengths. First, the inclusion of fathers in this study is fairly unique within studies exploring food parenting practices [13,28], or children’s health in general [29]. This allowed for a more accurate analysis of the context in which preschoolers from primarily two-parent families are fed. Second, the use of direct observation allowed us to more accurately explore associations between food parenting practices and children’s nutrition risk. Furthermore, these observations were conducted in the home, without the presence of an RA, to allow for typical mealtime interactions to occur. Many studies exploring food parenting practices have either been conducted in lab settings or in the home in the presence of an RA where interactions are more likely to be atypical. Third, the use of a validated nutrition risk screening tool also adds to the strength of our study, ensuring an accurate measure of nutrition risk. Finally, this study is the first to observe food parenting practices in a Canadian context, which allows us to better understand the mealtime environments in which Canadian preschoolers are fed.

Despite the many strengths, there are limitations that should be noted when interpreting our results. This study was cross-sectional, and thus, the bi-directional nature of parent-child feeding interactions could not be determined. For example, it is possible that more controlling feeding practices are used with picky eaters; parents may use these practices in an attempt to improve their child’s nutritional intake. Prospective research is needed to understand the temporal order of the association between food parenting practices and children’s dietary intake as well as the bi-directional nature of these feeding interactions. While families recorded three mealtimes, we only observed food parenting practices during the evening meal. It is possible that food parenting practices differ during less structured eating occasions such as snack time. The level of nutrition risk reported in this study was much lower than previous studies exploring NutriSTEP^®^ risk scores in Canada. Results may differ among families with children who have higher nutrition risk. Similarly, the families in this study also had high levels of family functioning. Results may also differ among families with lower functioning. While we found a significant association between mothers’ use of physical restriction and mothers’ and fathers’ positive comments about the target child’s food with NutriSTEP^®^ risk scores, it should be noted that the effect sizes of the associations were relatively small. We have NutriSTEP^®^ data from just one parent (primarily mothers) which may impact our findings and explain the few associations between fathers’ food parenting practices and NutriSTEP^®^ scores. Parents may perceive their child’s nutrition risk differently; future research should seek to understand differences in how mothers’ and fathers’ report their child’s nutrition risk. We calculated 36 tests (Table 4) and did not account for multiple comparisons. However, of these tests, five were statistically significant at the *p* < 0.05 level, which is larger than the one test we would expect by chance. Finally, the families in this study were highly educated and the majority identified as “white”; our results may not be generalizable to more diverse populations.

## 5. Conclusions

Mothers’ observed use of physical restriction was associated with increased nutrition risk and mothers’ and fathers’ use of positive comments about the target child’s food was associated with lower nutrition risk among preschool aged children. Family functioning did not moderate or confound the associations between parental food parenting practices and children’s nutrition risk. Results suggest that supporting parents to use more positive encouragement rather than restriction may help to reduce preschoolers’ nutrition risk. Future research should test interventions aimed at changing food parenting practices among families with preschoolers at medium-high nutrition risk. While we found few associations between fathers’ food parenting practices and preschoolers’ nutrition risk, we found differences among the types and frequency of food parenting practices employed by mothers and fathers. This underscores the importance of including fathers in food parenting research. As this was the first Canadian study to observe food parenting practices, future research is needed among more diverse populations, including those with more socio-economic diversity, higher levels of nutrition risk, and lower levels of family functioning, to further elucidate the association between food parenting practices and children’s nutrition risk in Canada.

## Figures and Tables

**Table 1:** Parent and family-level characteristics of participants in the Family Mealtime Observation Study (FaMOS) (*n* = 73 Families; 137 Parents).

**Parental Characteristics (*n* = 137)**	
	***n*** **(%)**
**Relation to Child**	
Mother ^i^	74 (54.0)
Father	63 (46.0)
**Parental Age in years, mean (SD)**	36.1 (9.5)
**Parental Educational Attainment**	
High School Education or Less	5 (3.7)
Some College or University	15 (11.0)
College Graduate	18 (13.2)
University Graduate	50 (36.8)
Post Graduate Training or Degree	48 (35.3)
**Parent Race/Ethnicity**	
White	115 (84.6)
Chinese	5 (3.7)
Latin American	6 (4.4)
Other (South Asian, Southeast Asian, West Indian, Black, Aboriginal/Indigenous)	10 (7.3)
**Parent Birth Country**	
Outside of Canada	18 (14.9)
**Parental Weight Status (BMI, kg/m^2^) ^ii^, mean (SD)**	**26.9 (6.5)**
Underweight (BMI <18.5)	1 (0.7)
Normal Weight (BMI 18.5–24.9)	56 (43.8)
Overweight/Obese (BMI ≥25)	71 (55.5)
**Family-Level Characteristics (*n* = 73)**	
	***n*** **(%)**
**Family Structure**	
Married or living with a partner	69 (94.5)
**Total Household Income**	
<$49,999/year	10 (13.9)
$50,000–$99,999/year	25 (34.7)
≥$100,000/year	37 (51.4)
**Family Functioning, mean SD**	**1.62 (0.39)**
High (<2.17)	66 (90.4)
Low (≥2.17)	7 (9.6)
**Family Dinners (days/week)**	
Every day (7 days/week)	61 (83.6)
Most days (4–6 days/week)	9 (12.3)
Rarely (<3 days/week)	3 (4.1)

^i^ One same-sex couple. ^ii^ Parent weight missing for pregnant mothers (*n* = 9). SD standard deviation; BMI: Body Mass Index.

**Table 2:** Characteristics of children participating in the Family Mealtime Observation Study (FaMOS) (*n* = 73).

*n* (%)	
**Child Age mean (SD) 3.3 years (1.1)**	
**Child Gender**	
Female	41 (56.2)
**Child Race/Ethnicity**	
White	57 (78.1)
Other (including Latin American, Southeast Asian, Chinese, Black and West Indian)	16 (21.9)
**Child Weight Status (BMI *z*-score) ^i^ mean (SD) 0.9 (1.9)**	
Healthy Weight	47 (65.3)
At risk of Overweight	19 (26.4)
Overweight/Obese	6 (8.3)
**NutriSTEP^®^ Score mean (SD) 13.3 (5.2)**	
Low risk (≤20)	65 (89.0)
Medium risk (21–25)	6 (8.2)
High risk (>25)	2 (2.7)

^i^ Child weight missing for one child who declined measurement. SD: standard deviation; BMI: Body Mass Index.

**Table 3:** Descriptive and frequency statistics for mothers’ and fathers’ observed food parenting practices ^a^ with target child and Mann-Whitney *U*-tests of difference between mothers’ and fathers’ observed food parenting.

	Mothers (*n* = 74)			Fathers (*n* = 63)			Mann-Whitney *U*-Test *z*-Scores
	Mean (SD)	Minimum	Maximum	Mean (SD)	Minimum	Maximum	
Verbal Pressure to eat	4.37 (5.22)	0	37.5	3.03 (3.06)	0	11.5	−1.7
Physical Pressure to eat	2.31 (4.22)	0	21	1.63 (2.80)	0	13	−0.67
Verbal Restriction	**1.31 (1.59)**	**0**	**9.5**	**0.63 (0.88)**	**0**	**4**	**−3.13**
Physical Restriction	0.21 (0.43)	0	2	0.12 (0.35)	0	2	−1.29
Use of Food as Reward	**0.75 (1.79)**	**0**	**14.5**	**0.49 (1.13)**	**0**	**6.5**	**−2.00**
Use of Non-Food Rewards	**0.31 (0.57)**	**0**	**2.5**	**0.14 (0.36)**	**0**	**2**	**−2.02**
Positive comments about food in general	3.91 (3.29)	0	14	3.11 (2.96)	0	16	−1.42
Positive comments about own food	1.83 (1.47)	0	7.5	1.78 (1.54)	0	5.5	−0.42
Positive comments about target child’s food	**7.12 (5.31)**	**0**	**25**	**4.26 (3.45)**	**0**	**14.5**	**−3.52**

^a^ Measured by the Family Mealtime Coding System. Significant differences at *p* < 0.05 level are bolded. SD standard deviation.

**Table 4:** Linear Regression models examining associations of Observed Food Parenting Practices with NutriSTEP^®^ Scores.

Observed Food Parenting Practice	Mothers (*n* = 74)		Fathers (*n* = 63)	
	Model 1	Model 2	Model 1	Model 2
	Effect Estimate (95% CI)		Effect Estimate (95% CI)	
Verbal Pressure to eat	−0.01 (−0.25, 0.22)	−0.01 (−0.24, 0.23)	0.03 (−0.37, 0.46)	0.04 (−0.36, 0.50)
Physical Pressure to eat	−0.05 (−0.36, 0.23)	−0.05 (−0.35, 0.24)	0.20 (−0.09, 0.80)	0.21 (−0.10, 0.80)
Verbal Restriction	0.28 (−0.68, 0.88)	0.30 (−0.68, 0.89)	0.13 (−0.73, 2.17)	0.15 (−0.67, 2.29)
Physical Restriction	**0.40** **(2.30, 7.58)**	**0.41** **(2.39, 7.72)**	0.03 (−3.00, 3.99)	0.06 (−2.88, 4.52)
Use of Food as Reward	−0.14 (−1.10, 0.26)	−0.14 (−1.10, 0.27)	−0.08 (−1.44, 0.79)	−0.08 (−1.45, 0.80)
Use of Non-Food Rewards	−0.16 (−3.59, 0.69)	−0.16 (−3.65, 0.67)	0.01 (−3.43, 3.64)	0.02 (−3.36, 3.79)
Positive comments about food in general	−0.16 (−0.63, 0.11)	−0.16 (−0.63, 0.11)	0.15 (−0.18, 0.67)	0.18 (−0.15, 0.73)
Positive comments about own food	−0.15 (−1.40, 0.29)	−0.15 (−1.40, 0.31)	−0.13 (−1.19, 0.42)	−0.14 (−1.24, 0.40)
Positive comments about target child’s food	**−0.31** **(−0.54, −0.08)**	**−0.31** **(−0.54, −0.08)**	**−0.27** **(−0.75, −0.01)**	−0.27 (−0.76, 0.00)

Dependent Variable: NutriSTEP^®^ Risk Score, continuous Model 1. Adjusted for parent educational attainment, dichotomous and child BMI-*z* score, continuous Model 2. Adjusted for model 1 covariates and family functioning, continuous Estimates where the 95% Confidence Interval (CI) does not include 0.0 are bolded.

## Data Availability

The datasets used and/or analyzed during the current study are available from the corresponding author on reasonable request.

## Abbreviations

FaMOS	Family Mealtime Observation Study
FMCS	Family Mealtime Coding Scheme
BMI	Body Mass Index
NutriSTEP^®^	Nutrition Screening Tool for Every Preschooler
WHO	World Health Organization
FAD	Family Assessment Device
